# Eating the messenger (RNA): autophagy shapes the cellular RNA landscape

**DOI:** 10.1093/jxb/erab385

**Published:** 2021-09-01

**Authors:** Girishkumar Kumaran, Simon Michaeli

**Affiliations:** Institute of Postharvest and Food Sciences, Agricultural Research Organization (ARO)-Volcani Institute, Rishon LeZion, Israel

**Keywords:** ATG5, autophagy, plant vacuole, RNAome, RNase, RNA-seq, RNS2

## Abstract

This article comments on:

**Hickl D, Drews F, Girke C, Zimmer D, Mühlhaus T, Hauth J, Nordström K, Trentmann O, Neuhaus EH, Scheuring D, Fehlmann T, Keller A, Simon M, Möhlmann T.** 2021. Differential degradation of RNA species by autophagy-related pathways in Arabidopsis. Journal of Experimental Botany **72**, 6867–6881.

**Cellular degradation pathways are instrumental for maintenance of homeostasis, especially under stress. Autophagy, the collection of pathways that shuttle cytoplasmic material to the vacuole for degradation and recycling, is known for shaping the cell’s proteome, lipidome, metabolome, and organelle content in response to environmental and developmental cues. However, until recently, little was known regarding its role in shaping cellular RNA quantity and diversity (RNAome). Recent data, including those from Hickl *et al.* (2021), highlight autophagy as a central pathway in RNA degradation, including the selective targeting of specific mRNA species, suggesting its role in post-transcriptional regulation.**

To maintain viability, all organisms have to balance biosynthesis and biodegradation pathways in a highly regulated fashion, especially under stressful conditions. One of the major cellular biodegradation pathways is autophagy, an evolutionarily conserved process that eliminates superfluous and impaired cellular components. Autophagy defines a collection of cellular pathways with the shared characteristic of displacing cytosolic cellular cargo into the cell’s lytic compartment (lysosomes in animal cells or vacuoles in yeast and plant cells). The two main mechanisms that operate in plants are macroautophagy and microautophagy (Box 1). The autophagy cargo that is destined for degradation may comprise entire organelles, organelle components, macromolecules, and invading pathogens (Marshall and Vierstra, 2018). Autophagy is carried out and regulated through the action of >30 different autophagy-related (ATG) proteins. Some of these, for example ATG5, are essential for autophagy in plants and other organisms (Thompson *et al.*, 2005). Autophagy may be highly selective for specific types of cargo, depending on the developmental or environmental context (Boxes 1, 2).

Box 1. **Microautophagy, macroautophagy, and selective autophagy in brief**

During macroautophagy, a double membrane structure is forming and building in proximity to the cytosolic cargo that is destined for degradation (termed the phagophore), until the full engulfment of the cargo (termed the autophagosome). Then, the outer autophagosome membrane fuses with the tonoplast (vacuole-delimiting membrane), releasing an autophagic body into the vacuole lumen for degradation. During microautophagy, the vacuole takes up a portion of the cytoplasm directly, generating a microautophagic body that will be digested within the vacuole. More than 30 autophagy-related (ATG) genes involved in macro- and microautophagy have been identified over the years. Nevertheless, microautophagy can be further divided into ESCRT (endosome sorting complex required for transport)-dependent and ATG-dependent pathways (Schuck, 2020).

Both micro- and macroautophagy pathways may demonstrate high selectivity for certain types of cargo, both in the frame of a housekeeping role and under stress. During selective autophagy, specific cargo is recognized via specialized proteins termed cargo receptors, and is taken up into the vacuole with the participation of the core autophagy proteins (Marshall and Vierstra, 2018).

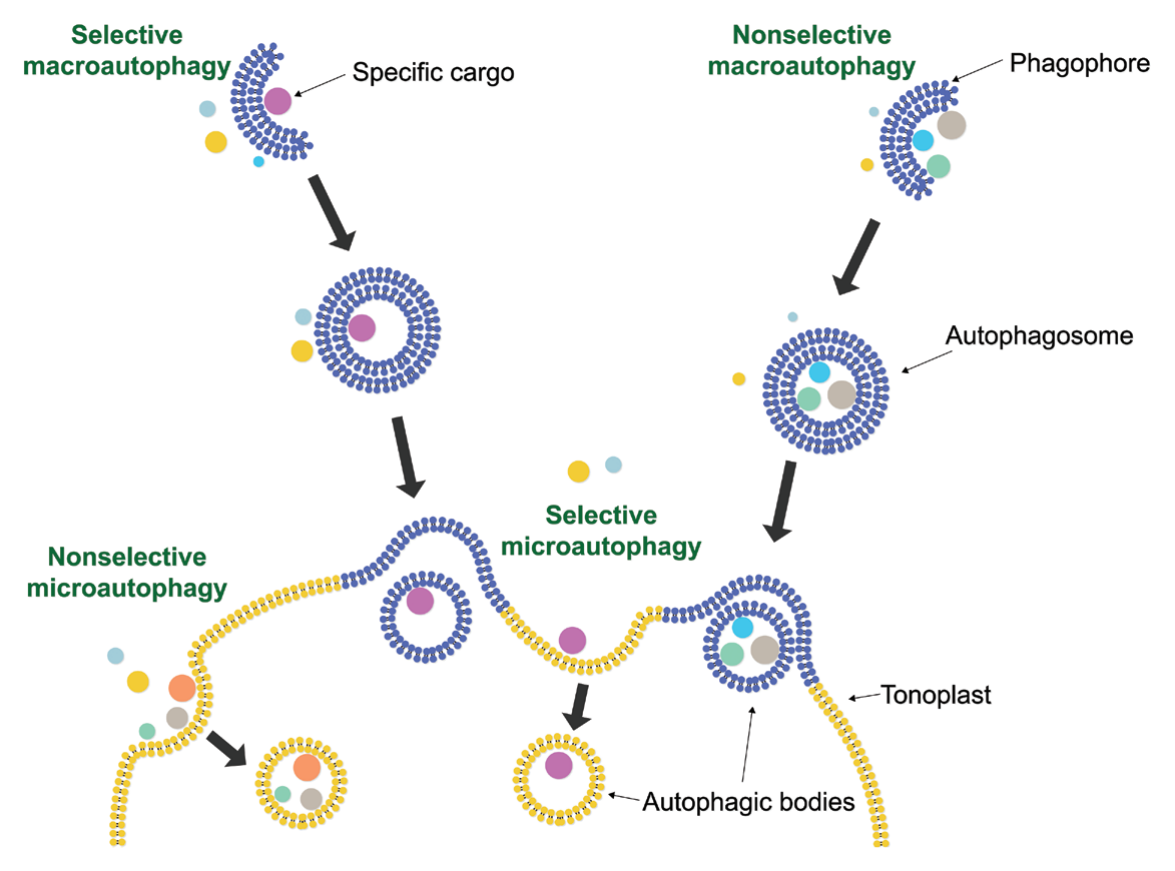

Box 2. **Possible sources and selective autophagy pathways for selective RNA degradation**

Selective autophagy pathways bear specific names, usually based on the combination of the cargo source name and the suffix ‘phagy’. For example, selective autophagy of mitochondria is termed ‘mitophagy’. Here we gathered several (not necessarily an exhaustive list) sources for selective RNA degradation and the selective autophagy pathways that may mediate them. Some of these may be the source of one particular type of RNA, while others may be the source of various RNAs. For example, nucleophagy may mediate the degradation of small RNAs, long non-coding RNAs, mRNAs, etc. Conversely, the selective autophagy of RNA-binding proteins may deliver one type of RNA to the vacuole. For example, AGO1, a central component of the RNA-induced silencing complex (RISC), whose degradation is mediated, at least partly, by selective autophagy (Michaeli *et al.*, 2019), might be a selective source of small RNAs. AGO1 is depicted in the figure while loaded with a small ssRNA.

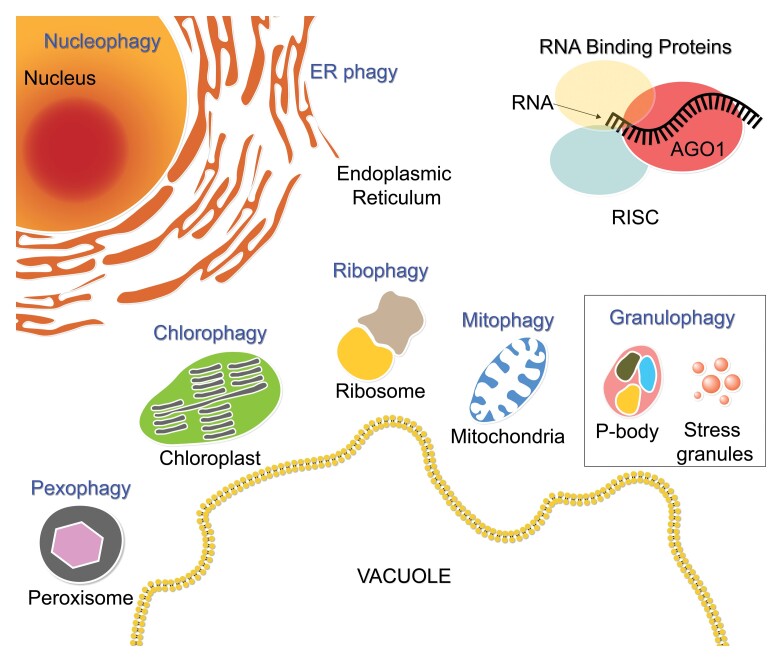

## RNA autophagy (RNAphagy)

RNA degradation is known to occur in the cell’s cytoplasm and nucleus through the RNA decay and mRNA surveillance pathways (Frankel *et al.*, 2017). However, whether RNA is delivered to vacuoles/lysosomes for degradation has been a long-standing question. Early reports suggested autophagy-dependent RNA degradation in human fibroblasts and amino acid-starved rat livers (Sameshima *et al.*, 1981; Lardeux and Mortimore, 1987; Balavoine *et al.*, 1990; Heydrick *et al.*, 1991). The subject was revived later following two reports on autophagy‐dependent RNA degradation. One described autophagy of rRNA in Arabidopsis (Floyd *et al.*, 2015), while the other described bulk autophagy of RNA under nitrogen starvation in yeast (Huang *et al.*, 2015). Intriguingly, vacuole-residing T2-type RNases were implicated in both cases. In Arabidopsis, RNS2 is essential for vacuolar RNA recycling, and *rns2* mutants exhibited constitutive autophagy during favourable growth conditions, under which autophagy is only slightly active in wild-type plants. The authors suggested that this as a compensatory mechanism for the lack of proper RNA degradation (Hillwig *et al.*, 2011). In yeast, RNA that reaches the vacuole is degraded by a single vacuolar RNase, Rny1 (Huang *et al.*, 2015), a functional homologue of plant RNS2 (MacIntosh *et al.*, 2001). However, its deficiency does not seem to induce autophagy (Huang *et al*, 2015).

Ultimately, two main questions remained unanswered. (i) Is RNAphagy a bulk or selective process? (ii) Is autophagy involved in shaping the cellular RNAome under changing environmental conditions?

## Selective RNA autophagy (RNAphagy)

Initial reports addressing these questions emerged recently. First, Makino *et al.* (2021) profiled mRNA from vacuoles of yeast Rny1 cells following autophagy induction through target of rapamycin (TOR) inhibition. They detected enrichment of certain mRNAs, especially those encoding amino acid biosynthesis and ribosomal proteins. Furthermore, ribosome profiling suggested high correspondence between ribosome–mRNA association (polysomes) and the identity of the vacuole-residing mRNAs. Nevertheless, selective autophagy of ribosomes (ribophagy) and the endoplasmic reticulum (ER-phagy) (Box 2) are not the routes implicated in polysome degradation (Makino *et al.*, 2021). Notably, as TOR is also known as a translation regulator, it is reasonable to expect that enrichment of different mRNA species in yeast vacuoles will occur under different autophagy-inducing conditions (especially those that do not rely on TOR inhibition). Taken together, the authors proposed autophagy as a post-transcription regulator (Makino *et al.*, 2021). A similar suggestion was raised after identifying a plant selective ER-phagy pathway that clears ER-bound Argonaute 1 (AGO1; Michaeli *et al.*, 2019), which is apparently associated with membrane-bound polysomes and acts in inhibition of translation (Li *et al.*, 2013). Consistently, an Arabidopsis mutant deficient in this pathway exhibited a significantly reduced post-transcriptional gene silencing (PTGS) activity (Michaeli *et al.*, 2019).

Hickl *et al.* (2021) also addressed the questions above by profiling vacuole RNAomes of Arabidopsis leaf mesophyll cells and comparing them with cytosolic RNAomes in the wild type and *rns2* and *atg5* mutants. However, while Makino *et al*. induced autophagy prior to vacuole profiling, Hickl *et al*. profiled vacuoles of plants experiencing favourable growth conditions, hence exhibiting low autophagy activity. Unexpectedly, RNS2 deficiency showed a relatively mild difference in the RNAome profile compared with the wild type, whereas ATG5 deficiency showed a more profound effect. Mainly chloroplast-encoded mRNA transcripts were significantly under-represented in *atg5* vacuoles, whereas nuclear-derived transcripts encoding photosynthesis-associated proteins were found enriched in this mutant (Hickl *et al.*, 2021). These differences, mainly in photosynthesis-related genes, highlight an important role for autophagy in chloroplast turnover, even under favourable conditions. Notably, this work shows that differential delivery of specific mRNAs to vacuoles also occurs in plants. Although the source of chloroplast-encoded mRNA found in the vacuole is unknown, one can assume that selective autophagy of chloroplast components or the selective autophagy of entire chloroplasts (Otegui, 2018; Box 2) is implicated in delivering this type of molecule to the vacuole (potentially with additional cargo). Indeed, both pathways rely on functional ATG5 proteins.

Regarding the counterintuitive enrichment of nuclear-encoded chloroplast-related transcripts, Hickl *et al*. suggest the induction of a compensatory, ATG5-independent, autophagy pathway as a possible explanation. Such a pathway, targeting chloroplast components, was identified in plants (Wang and Blumwald, 2014). However, in Hickl *et al*., such an alternative pathway supposedly targets the nucleus or perhaps cytosolic polysomes, for which alternative autophagy is not yet described in plants. Mammalian macroautophagy that operates independently of Atg5 and Atg7 was described (Nishida *et al.*, 2009). If such a pathway exists in plants, then its particular preference for photosynthesis-related mRNAs is intriguing. The modest impact of RNS2 deficiency is also surprising, yet is explained by redundancy with other potential vacuole-residing RNases, whereas it seems that Rny1 represents the only vacuolar yeast RNase (Makino *et al.*, 2021).

Nevertheless, it would be fascinating to examine the Arabidopsis vacuolar RNAome in both the wild type and *rns2* following autophagy induction. Will RNS2 make a difference when coupled to autophagy activity, especially when considering that *rns2* mutants were reported to have an endogenously high autophagy activity (Hillwig *et al.*, 2011)?

Equally intriguing, Hickl *et al*. also identified complete and mature miRNAs within the vacuole, suggesting that vacuoles act as an additional small RNA reservoir. Several sources for vacuolar miRNA may include the ER, nuclei, P-bodies, stress granules, peroxisomes, and miRNA-binding proteins (Box 2).

## What is next?

It is reasonable to assume that the intensity and selectivity of autophagy, which rely on the cell type, environmental conditions, and developmental state, will determine the targeted RNA’s identity and quantity. Moreover, the post-transcriptional and post-translational regulation of ATG mRNAs and proteins (Abildgaard *et al.*, 2020) may suggest a regulatory feedback loop between autophagy and RNA. Both Hickl *et al*. and Makino *et al.* describe a snapshot of the vacuolar RNAome at a particular time under a specific condition. Future studies that will examine the flux of different RNA species to the vacuole under other conditions will shed more light on this intriguing aspect of autophagy. Yet, it already seems that the differential activation of selective autophagy pathways to target RNA-containing cargo (Box 2) is a significant factor in shaping the cellular RNAome (including the mRNAome), thus affecting cellular gene expression post-transcriptionally.
